# Genetics Notes: A new category for descriptive genetics work

**DOI:** 10.1002/ece3.10992

**Published:** 2024-02-22

**Authors:** Gareth B. Jenkins, Andrew P. Beckerman, Allen J. Moore, Alison G. Nazareno, Chris Cunningham

**Affiliations:** ^1^ John Wiley & Sons Ltd Oxford UK; ^2^ School of Biosciences, Ecology and Evolutionary Biology University of Sheffield Sheffield UK; ^3^ Department of Entomology University of Georgia Athens Georgia USA; ^4^ Department of Genetics, Ecology and Evolution Federal University of Minas Gerais Belo Horizonte Brazil

One of the founding principles of *Ecology & Evolution* was to be author‐friendly; to work with researchers to get their work published irrespective of novelty or “impact” (Moore, 2011). This remains the key focus of our work as editors. However, we also aim to be innovative, through initiatives such as introducing new article types or adopting open science practices. These initiatives are primarily driven by our author community; if we see a large number of submissions coming in that are out of scope for our current framework, then the onus is on us to see what we can do to accommodate them.

Over the last couple of years, we have seen an increase in submissions of descriptive genetics material. These “notes” are not hypothesis‐driven and as such, do not fall under any of our current article types. We often find we have to reject them unless they are merely part of a more holistic study (e.g., phylogenetic analyses). However, there is often a lot of research effort that goes into generating these data as they represent the first step of larger projects many times, and there is certainly value in them as a reference library to inform further research in fields such as population or conservation genetics. Instead of contributing to this wider resource, authors are increasingly having to shoehorn their data into an acceptable format rather than publishing simply what they wish to publish. We believe that there is a lack of journals that address this need, over the broad scope and range of fields we cover. So, we are looking to fix that by introducing a new article type, *Genetics Notes*.

These notes will serve as a sister category to our descriptive natural history article type, *Nature Notes* (Jenkins et al., 2022; Moore et al., 2020), and be evaluated purely on the soundness of the methodology rather than as a more conventional research article. If the editors believe that the genetic data reported will have value to our community, we will consider it. We will not dictate any set format; authors should report their work as they see fit for peer review. However, authors should be aware of any general reporting guidelines and quality thresholds that may already exist for their work and adhere to them (e.g., MINSEQE, MIAPE).

The following list is example of the types of work we will consider, but is not exhaustive and should be taken as an indication only:

1. Organellar and nuclear (pan)genome assemblies or significant re‐assemblies
2. Single or multiple taxa (pan)transcriptome assemblies
3. Single cell atlases
4. Identification of DNA barcode markers and development of reference libraries
5. Environmental DNA (eDNA) sample reports
6. Proteomic or Metabolomic sample reports
7. Single Nucleotide Polymorphism (SNP) and Structural Variants (SV) reports
8. Development and validation of SNP arrays, probe sets or sequence‐capture baits
9. Characterization of novel nuclear and chloroplast microsatellite markers
10. Optimization of methods for whole‐genome and reduced representation sequencing
11. Innovative molecular methods

Our usual data policy will apply to *Genetics Notes*, as with all our other article types, that is, authors are mandated to supply data at submission which comply with our minimum standards (Jenkins et al., 2023). Unless there are specific restrictions that thwart open access data sharing, short‐ and long‐read raw data, novel nucleic acid sequences, organellar and nuclear genome assemblies, proteomic and (functional) annotated sequences should be deposited to the most appropriate partners of the International Nucleotide Sequence Database Collaboration (NCBI, DDBJ, or EMBL‐EBI). Genetic variation datasets must also be deposited in a long‐term accessibility repository (e.g., Data Science Bank, Dryad, European Variation Archive, FigShare, Open Science Framework, and Zenodo). For DNA Barcode studies, sequences should be deposited in the Barcode Life Data System (BOLD).

## AUTHOR CONTRIBUTIONS

**Andrew P. Beckerman:** Conceptualization (supporting); writing – original draft (supporting); writing – review and editing (supporting). **Allen J. Moore:** Conceptualization (supporting); writing – original draft (supporting); writing – review and editing (supporting). **Alison G. Nazareno:** Writing – original draft (supporting); writing – review and editing (supporting). **Chris Cunningham:** Writing – original draft (supporting); writing – review and editing (supporting). **Gareth B. Jenkins:** Conceptualization (lead); writing – original draft (lead); writing – review and editing (lead).

## Data Availability

No data generated—this is an editorial.
